# Supplementary material for: End Sequence Analysis Toolkit (ESAT) expands the extractable information from single-cell RNA-seq data
Source: Genome Res. 2016 Oct;26(10):1397–410. doi: 10.1101/gr.207902.116 (PMC5052061; doi:10.1101/gr.207902.116)
Supplement: Supplemental Material [file supp_gr.207902.116_Supp_Materials.tar.gz › ESAT-esat_v0.1/doc/html/classumms_1_1core_1_1read_filters_1_1_read_starts_in_filter.html]

ESAT: umms.core.readFilters.ReadStartsInFilter Class Reference


ESAT


- Main Page
- Related Pages
- Packages
- Classes
- Files

- Class List
- Class Index
- Class Hierarchy
- Class Members

All Classes Namespaces Files Functions Variables Enumerator Pages

- umms
- core
- readFilters
- ReadStartsInFilter

Public Member Functions |
List of all members

umms.core.readFilters.ReadStartsInFilter Class Reference

Inheritance diagram for umms.core.readFilters.ReadStartsInFilter:

[legend]

Collaboration diagram for umms.core.readFilters.ReadStartsInFilter:

[legend]

|  |  |
| --- | --- |
| Public Member Functions | |
|  | ReadStartsInFilter (Annotation w) |
|  | |
| boolean | evaluate (Alignment read) |
|  | |

## Constructor & Destructor Documentation

|  |  |  |  |  |  |
| --- | --- | --- | --- | --- | --- |
| umms.core.readFilters.ReadStartsInFilter.ReadStartsInFilter | ( | Annotation | *w* | ) |  |

## Member Function Documentation

|  |  |  |  |  |  |
| --- | --- | --- | --- | --- | --- |
| boolean umms.core.readFilters.ReadStartsInFilter.evaluate | ( | Alignment | *read* | ) |  |

Here is the call graph for this function:

---

The documentation for this class was generated from the following file:

- java/umms/core/readFilters/ReadStartsInFilter.java


---

Generated on Fri Aug 15 2014 15:11:14 for ESAT by  

 1.8.7
